# Airport and luggage (Odyssean) malaria in Europe: a systematic review

**DOI:** 10.2807/1560-7917.ES.2024.29.41.2400237

**Published:** 2024-10-10

**Authors:** Luisa K Hallmaier-Wacker, Merel D van Eick, Olivier Briët, Hugues Delamare, Gerhard Falkenhorst, Sandrine Houzé, Harold Noël, Javiera Rebolledo, Wim Van Bortel, Céline M Gossner

**Affiliations:** 1 Epidemic Prone Disease Section, European Centre for Disease Prevention and Control (ECDC), Stockholm, Sweden; 2 Department of Public and Occupational Health, Amsterdam Public Health Research Institute, Amsterdam University Medical Centers, Free University of Amsterdam, Amsterdam, the Netherlands; 3 Santé publique France, Saint-Maurice, France; 4 Department of Infectious Disease Epidemiology, Robert Koch Institute, Berlin, Germany; 5 Université de Paris Cité, MERIT, IRD, Paris, France & Centre National de Référence du Paludisme, Hôpital Bichat, AP-HP, Paris, France; 6 Service of Epidemiology of infectious diseases, Sciensano, Brussels, Belgium; 7 Unit Entomology, Department of Biomedical Sciences, Institute of Tropical Medicine, Antwerpen, Belgium; 8 Outbreak Research Team, Department of Biomedical Sciences, Institute of Tropical Medicine, Antwerpen, Belgium

**Keywords:** malaria, airport, luggage, Odyssean malaria, *Plasmodium* spp., *Anopheles* spp

## Abstract

**Background:**

Airport and luggage (also called Odyssean) malaria are chance events where *Plasmodium* infection results from the bite of an infected mosquito which was transported by aircraft from a malaria-endemic area. Infrequent case reports and a lack of central data collection challenge a comprehensive overview.

**Aim:**

To update the epidemiological, clinical and biological understanding of airport and luggage malaria cases in Europe.

**Methods:**

We conducted a systematic review of studies indexed from 1969 to January 2024 in MEDLINE, Embase and OpenGrey databases. A data call to EU/EEA and UK public health institutes was launched in December 2022.

**Results:**

Of the 145 cases (89 cases from 48 studies and 56 cases from the data call) described from nine countries, 105 were classified as airport malaria, 32 as luggage malaria and eight as either airport or luggage malaria. Most airport malaria cases were reported in France (n = 52), Belgium (n = 19) and Germany (n = 9). Half of cases resided or worked near or at an international airport (mean distance of 4.3 km, n = 28). Despite disruptions in air travel amid the COVID-19 pandemic, one third of cases reported since 2000 occurred between 2018 and 2022, with a peak in 2019.

**Conclusion:**

While airport and luggage malaria cases are rare, reports in Europe have increased, highlighting the need for effective prevention measures and a more structured surveillance of cases in Europe. Prevention measures already in place such as aircraft disinsection should be assessed for compliance and effectiveness.

## Introduction

Malaria was eradicated from western Europe in the 1970s and since then, most cases occurring in the European Union/European Economic Area (EU/EEA) are among travellers returning from malaria-endemic countries. Of the 6,131 cases reported in the EU/EEA in 2022 with known importation status (notification rate of 0.78 cases per 100,000 population), over 99% of cases were travel related [1]. Instances of locally acquired malaria within Europe are infrequent and can be categorised into three main types: (i) introduced cases resulting from transmission by an indigenous mosquito infected by an imported case, (ii) induced cases where malaria is not contracted through a mosquito bite, and (iii) airport/luggage malaria [2].

Airport malaria describes a *Plasmodium* infection, acquired at or near an airport, resulting from a bite from an infectious mosquito which was transported by aircraft from a malaria-endemic area. Luggage malaria is further distinguished, whereby the infectious mosquito is transported in baggage and released at a site of infection away from an airport. In practice, the classification of airport and luggage malaria is often made in malaria non-endemic areas, when there are no apparent epidemiological links other than proximity or link to an airport or a travel/luggage. A review from 1989 describing 29 cases of airport and luggage malaria in Europe from 1969 to 1988 highlights their sporadic occurrence [3]. Since then, cases of airport and luggage malaria have continued to be reported in Europe [4], with infrequent reports and the absence of centralised reporting challenging a comprehensive overview.

This systematic review aims to update the epidemiological, clinical and biological understanding of airport and luggage malaria cases in Europe through a review of the literature and a data call to European public health institutes. To support the interpretation of the results, we assessed the recent flight patterns between EU/EEA and malaria-endemic countries.

## Methods

This systematic review was conducted according to the recommendations of the Preferred Reporting Items for Systematic Reviews and Meta-Analyses (PRISMA) [5] and registered with PROSPERO (CRD42021248756).

### Search strategy

The initial search was conducted on 10 May 2021 and subsequently updated on 1 January 2024. Search terms for malaria: ‘malaria’, ‘plasmodium’, ‘falciparum’, ‘ovale’, ‘vivax’, ‘knowlesi’, ‘malariae’ were used in conjunction with terms on airport and luggage: ‘airport’, ‘luggage’, ‘suitcase’, baggage’, ‘aircraft’, ‘airline’, ‘plane’. No publication date restriction and no language restrictions were imposed. The following databases and libraries were searched: Ovid-Medline, Embase.com, Scopus, PubMed, OpenGrey and EBSCO open dissertations. The full search strategy for each database is provided in Supplementary Table S1.

### Screening

Citations identified by the search were imported into EndNote (EndNote X9, Clarivate Analytics, Boston, United States (US)) for de-duplication, and then imported into the automated tool Rayyan [6] for review. Two reviewers independently conducted a first screen by title and abstract (LKHW and MDE). Articles were included if: (i) cases were identified as airport or luggage malaria by the investigators, (ii) the investigators stated that *Plasmodium* was confirmed by any laboratory method, and (iii) the infection occurred in an EU/EEA country, the United Kingdom (UK) or Switzerland. Articles were excluded if: (i) the infection occurred in the EU outermost regions, and (ii) the original investigators concluded that the most likely transmission route was not airport or luggage malaria (e.g. related to blood products, indigenous *Anopheles* mosquitoes, or recent travel/layover to a malaria-endemic country). Additionally, references of review articles were screened to find publications not identified through the literature search. Full texts of all eligible articles were retrieved and independently screened for final inclusion by two reviewers (LKHW and MDE). Disagreements during screening were resolved by consensus together with a third reviewer (CMG).

### Quality assessment and data extraction

The methodological quality of all included studies was evaluated by one reviewer and subsequently checked by another reviewer using a quality assessment tool adapted from the United States National Institutes of Health Quality Assessment Tool for Case Series Studies (Supplementary Table S2) [7]. The adaptation of this tool allowed for the quality assessment of case reports, which represented most articles identified in the search. Studies were rated as ‘good’, ‘fair’ or ‘poor’ quality depending on their overall numeric rating. No studies were excluded based on the quality assessment. Data from all studies were subsequently extracted by one reviewer (MDE) and checked for accuracy and completeness by another reviewer (LKHW). The extracted data (Supplementary Table S3) were reviewed by three reviewers to remove duplicate case reports (MDE, LKHW, CMG).

### Other data sources

In December 2021 and 2022, a request for data was sent to all public health authorities of EU/EEA countries and the UK. The data records identified through the search pertaining to each country were sent in addition to a data sheet for additional cases. Public health authorities were requested to check for duplicates and submit any additional cases. The variables included in the data sheet and the list of cases are displayed in the Supplementary methods and Supplementary Table S3.

### Data analysis

We performed a descriptive analysis of identified case reports. Temporal analyses were based on the earliest time point linked to each case as described by each publication (e.g. date of symptom onset, date of admission). Two-sided t-tests were performed to compare age between outcome (died vs recovered) and link to the airport (occupational link). Two-sided t-tests were performed to compare distance to airport between luggage and airport malaria cases (significance level of 0.05). All analyses were performed using the tidyverse package in R software (version 4.0.2) [8]. Maps were produced using ArcGIS software version Pro 3.0.3 (ESRI, Redlands, US).

To support the interpretation of the results of this systematic review, we examined the flight volume between malaria-endemic countries and airports in the EU/EEA. We analysed EUROCONTROL (international aviation organisation comprised of countries in Europe) flight traffic data (all air traffic including cargo flights, EUROCONTROL 2024) from January 2021 to December 2022. The time frame of the EUROCONTROL analysis was chosen on the basis of data availability. All countries classified for the purpose of this analysis as having a high incidence of *P. falciparum* cases were in the African continent (arbitrary threshold used for this study was greater than 70 *P. falciparum* cases per 1,000 population in 2020 as reported by the Malaria Atlas Project Data Platform [9]). Detailed methods of the flight pattern analysis are outlined in the Supplementary material.

## Results

**Figure 1:**
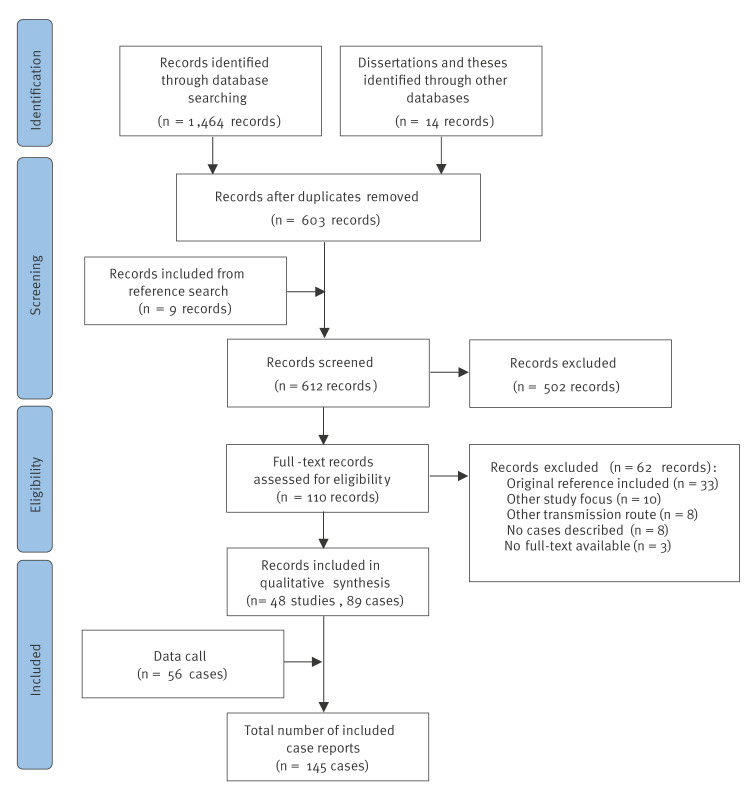
Preferred Reporting Items for Systematic Reviews and Meta-Analyses (PRISMA) flow diagram for study selection

Database searches identified 612 unique records (Figure 1), of which 110 were retained for full-text review. Following the application of the exclusion criteria, 48 studies describing 89 cases of airport or luggage malaria were included for data qualitative assessment and data extraction [10-57]. Of the 48 studies included for data extraction, 20 were rated good [11,17,18,20,24-27,32,34,36-38,41-44,46,53,56], 18 fair [10,12-14,22,23,29-31,35,39,40,45,47,50-52,55] and 10 as poor quality [15,16,19,21,28,33,48,49,54,57] further details on scored criteria are available in Supplementary Table S2. The quality of the studies improved in studies published after 2000 compared with studies published before. An additional 56 cases from six countries were provided in response to the data call [58], resulting in 145 included unique case reports. No quality assessment was conducted for the 56 cases identified through the data call.

**Figure 2:**
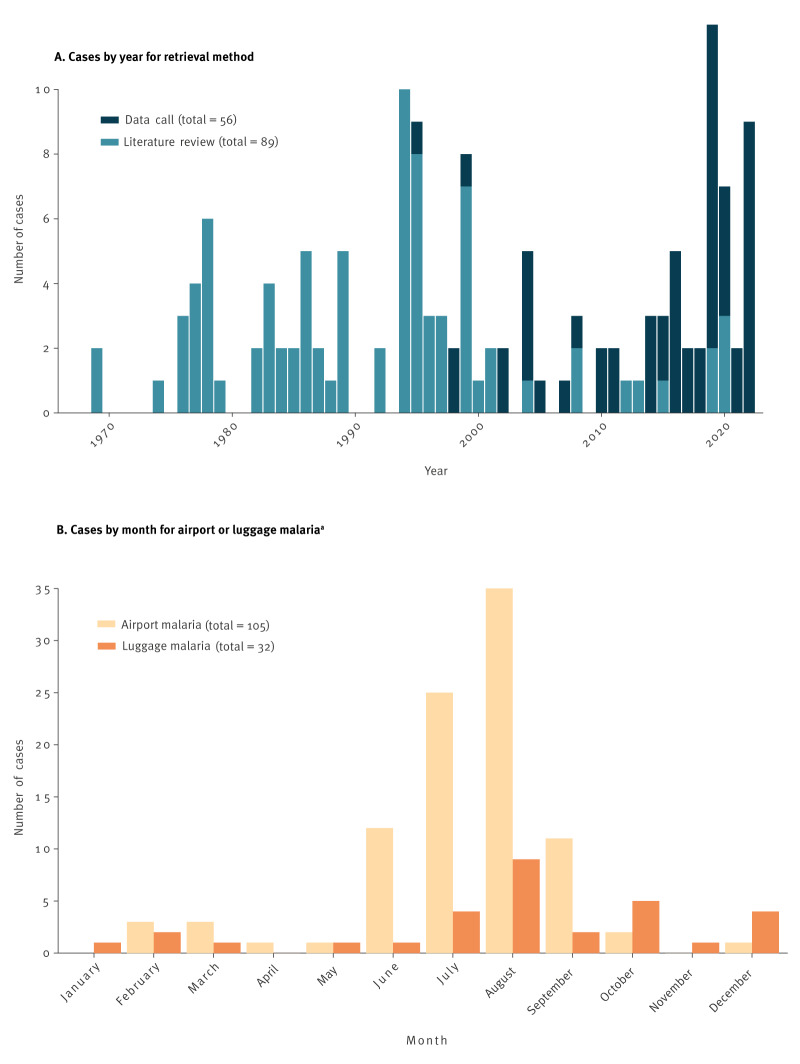
Number of cases by (A) year for the data retrieval method and (B) month for luggage and airport malaria, European Union/European Economic Area countries, Switzerland and the United Kingdom

^a^ Number of cases for European Union/European Economic Area countries and the United Kingdom obtained through literature review and data call; numbers for Switzerland obtained through literature review only.

Of the 145 cases identified, 105 were classified as airport malaria, 32 as luggage malaria and for eight cases, the investigators did not differentiate between airport and luggage malaria. The first case of airport malaria identified through our search was described in 1969 [17] followed by the first described occurrence of luggage malaria in 1974 [41]. Since then, airport and luggage malaria cases have been described sporadically, with most published case reports occurring in 1994 (n = 10) [35,42,43,55]. There has been a declining trend over the past 20 years of airport and luggage malaria cases described in peer-reviewed publications. However, data collected from public health agencies show that cases continue to be reported, with one third of cases since 2000 reported between 2018 and 2022 (Figure 2A).

The mean age of cases was 37.9 years (standard deviation (SD): ± 17.5) and cases were more likely to be male than female (sex ratio of 1.5:1, distribution of age and sex in Supplementary Figure S1A). Of 133 cases with known outcome, 93.2% (n = 124) recovered and 6.8% (n = 9) died. Patients that died were older with a mean age of 57.2 years (SD: ± 15.3, p = 0.008). Epidemiological links between cases were common with 48 of 145 cases epidemiologically linked to at least one other cases (a total of 18 clusters with a size of 2 to 6 cases per cluster), such as a common occupational exposure (n = 20) [34,42,43,47,55,57,58], co-habitation (n = 16) [17,19,21,24,36,38,53], residing near each other (e.g. neighbours) (n = 5) [23,32] and airport visits on the same day (n = 7) [47] (Supplementary Table S3).

Travel to or stay in a malaria-endemic country within 1 year of the infection was clearly described in a case of *P. falciparum* who had travelled to Indonesia 11 months prior. Lucania et al. concluded that the case was most likely airport malaria as the individual had followed adequate prophylaxis during the travel and there was a strong epidemiological link to two airport malaria cases [23]. Two additional cases were described as having stayed or travelled to a malaria-endemic country within the last 1–3 years [38,52]. Revel et al. described a case in a 7-year-old child who had resided in a malaria-endemic area approximately 3 years before the malaria infection. At the time of the study, the length of persistence of asymptomatic *P. falciparum* was thought to be less than 2 years [59], and the authors therefore concluded that luggage malaria was most likely. Bouvier et al. described a case in a former pilot with missions to potentially malaria-endemic countries. The case was linked with four other cases in Geneva airport. For three additional cases, the authors reported stay or travel to a malaria-endemic country within the past 6–20 years, with limited information on the stay/travel provided in the studies [11,18,46].

All cases for which information on diagnostic method was available were diagnosed by microscopy (n=122). For 25% of these cases (n=31), at least one complementary diagnostic test was performed: antibody testing, antigen detection and nucleic acid amplification. The mean delay in diagnosis was 7.5 days (range: 1–100 days, n = 67). A single *Plasmodium* species was identified in all but three cases, which was predominantly *P. falciparum* (n = 128). Of 145 cases with known species, *P. ovale* (n = 4) [47,56] (Supplementary Table S3), *P. vivax* (n = 3) [22,30,49] and *P. malariae* (n = 3) [58] infections were rarely reported. Co-infection with two *Plasmodium* species was also rare and always involved *P. falciparum* in combination with another species, either *P. vivax* (n = 2) [36], *P. ovale* (n = 1) [52] or *P. malariae* (n = 1) [23].

No case report identified infectious mosquitos in airports or luggage.

### Airport malaria

Of the 105 cases of airport malaria, 83 cases occurred between June and September, with a peak in August (Figure 2B). Eight cases were described outside this range, with three cases described in February (two cases infected in a hospital 3 km from the airport [36], one with an occupational link to the airport [46]) and one in December with unknown link to the airport (Supplementary Table S3)). For 11 cases, no month of infection was specified.

**Figure 3:**
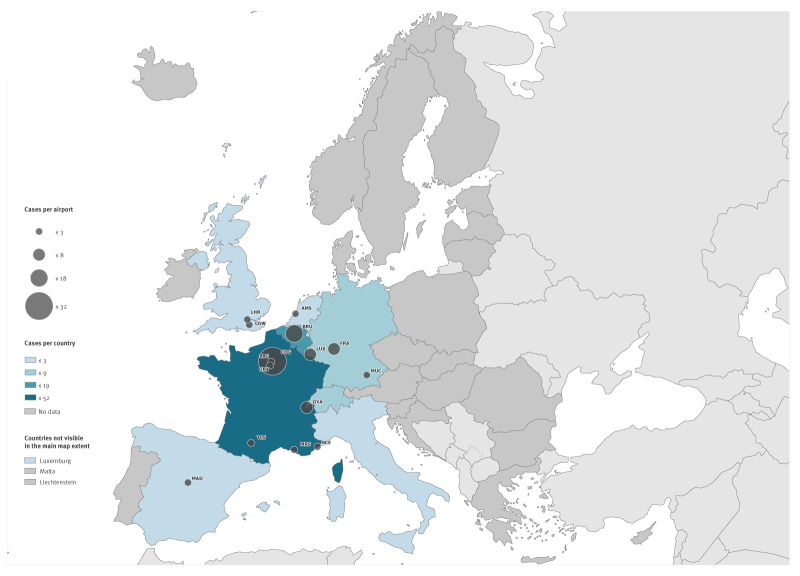
Map of airport malaria cases by airport^a^ and country, European Union/European Economic Area countries, Switzerland and the United Kingdom^b^

^a^ Amsterdam airport Schiphol (AMS, n = 3), Brussels airport (BRU, n = 18), Charles de Gaulle airport (CDG, n = 32), Frankfurt airport (FRA, n = 8), Geneva airport (GVA, n = 5), Paris–Le Bourget airport (LBG, n = 3), London Gatwick airport (LGW, n = 2), London Heathrow airport (LHR, n = 2), Luxembourg airport (LUX, n = 5), Madrid–Barajas airport (MAD, n = 1), Marseille Provence airport (MRS, n = 2), Munich airport (MUC, n = 1), Nice Côte d'Azur airport (NCE, n = 1), Paris-Orly airport (ORY, n = 3), Toulouse Blagnac airport (TLS, n = 2).

^b^ Number of cases for European Union/European Economic Area countries and the United Kingdom obtained through literature review and data call; numbers for Switzerland obtained through literature review only.

All cases of airport malaria were reported in western Europe, with most cases reported in France (49.5%, n = 52), followed by Belgium (18.1%, n = 19) and Germany (8.6%, n = 9) (Figure 3). Six airports in France were associated with airport malaria cases, more than in any other country. Paris Charles de Gaulle airport reported most cases (n = 32), with Marseille Provence airport, Nice Côte d'Azur airport, Paris–Le Bourget airport, Paris-Orly airport and Toulouse-Blagnac airport associated with sporadic case reports (Figure 3). Brussels airport was the airport with the second highest number of cases (n = 18), followed by Frankfurt airport (n = 8), Geneva airport (n = 5) and Luxembourg airport (n = 5). Three studies described the possibility of onward transport of the infectious mosquito in a car from the airport [32,42,45]. The mean distance to the closest airport for the five cases indicating onward transport by car was 9.3 km (range: 7–13 km).

The hypothesised link to an airport was described in most case reports (n = 97). Most often, cases resided or worked near an airport (38.1%, n = 40) with a mean distance of 4.3 km (range: 1–32 km, n = 28). A direct occupational link to an airport was described for 30 cases and 16 cases visited an airport or its proximate surroundings for a transient period (e.g. for picking up travellers). Cases with an occupational link to an airport were mostly working in occupations with close contact with the cargo or baggage hold (e.g. customs officer (n = 7) [15,22,57], baggage handler (n = 6)) [33,37,54] (Supplementary Table S3) and were predominantly male (sex ratio (M:F) 24:1, 5 cases reported no sex data, distribution of age and sex for individuals working at the airport is shown in Supplementary Figure S1B). The mean age of individuals with an occupation linked to an airport did not differ from other identified cases (39.3 years ± 10.8, p = 0.95).

### Luggage malaria

Case reports of luggage malaria have been sporadic with no more than three cases reported per year (n = 32 over the entire study period). Cases have been described throughout the year with 20 cases reported between July and October (Figure 2B). Two thirds of luggage malaria cases were reported in France (n = 23), followed by Italy (n = 3) and Germany (n = 3). Nine case reports mention a link to a specific airport, with Paris Charles de Gaulle airport most often mentioned (n = 5, see map of luggage malaria cases by airport and country shown in in Supplementary Figure S2). The mean distance to the closest airport was 42.5 km (range: 20–100 km, n = 6), significantly further than distances reported for airport malaria cases (p < 0.001).

### Flight patterns analysis

Between January 2021 and December 2022, a total of 48,296 direct flights (including commercial flights, cargo flights, helicopter flights, flights by private planes) from malaria-endemic countries landed at an airport within the EU/EEA. Among the high incidence malaria countries (70 *P. falciparum* cases per 1,000 population in 2020), Senegal, Nigeria and Ghana had the highest number of flights leaving to the EU/EEA (most frequent departure country shown in Supplementary Table S4). During the 2 years studied, France had the highest number of direct flights arriving from malaria-endemic countries (n = 15,382), followed by Belgium (n = 9,982) and Portugal (n = 6,492, Supplementary Table S5).

**Figure 4:**
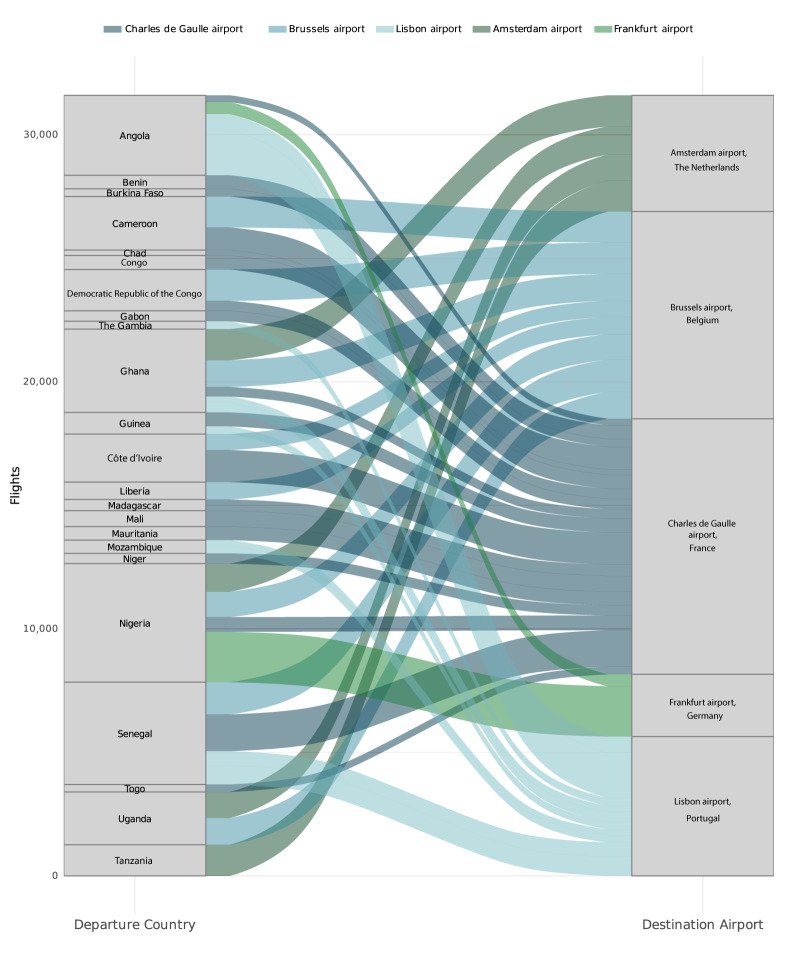
Flights between countries with malaria endemicity^a^ and the top five destination airports in the European Union/European Economic Area for 2021 and 2022

^a^ The arbitrary threshold used for this study was greater than 70 *P. falciparum* cases per 1,000 population in 2020 as reported by the Malaria Atlas Project Data Platform [59]).

Paris Charles de Gaulle airport and Brussels airport were the airports with the greatest number of flights arriving from malaria-endemic countries and were connected to more malaria-endemic countries by direct flights than any other airport in the EU/EEA (number of direct flights arriving to top destination airports in the EU/EEA shown in Supplementary Table S5, Figure 4). Other airports such as Frankfurt airport and Amsterdam airport had fewer direct flights originating from a smaller number of malaria-endemic countries with only one or two connections driving most of the observed traffic (e.g. connection Nigeria to Frankfurt airport driving 79% of traffic, Figure 4).

We selected for analysis the top five destination airports in the EU/EEA for 2021 and 2022. Only direct connections with more than 200 flights over the entire time period are shown. EUROCONTROL 2024 owns the copyright of the flight data used in this figure.

Overall, there was more traffic in 2022 compared with 2021 (temporal trends of the top five destination airports in the EU/EEA per month are shown in Supplementary Figure S3).

## Discussion

Instances of airport and luggage malaria in Europe are infrequent, yet there appears to be a rising trend in case reports over the past 5 years (2018–2022). This study reveals that more than a third of reported cases since 2000 occurred between 2018 and 2022, with a peak in 2019. It's noteworthy that recent cases were primarily identified through a data call to European national public health agencies, emphasising that not all documented cases are published. Therefore, caution is warranted when interpreting case counts solely based on those reported in peer-reviewed publications.

Several factors may contribute to the observed increase, including changes in aircraft disinsection practices, favourable climatic conditions near airports in Europe, heightened public awareness and changes in direct air traffic to malaria-endemic countries [4,30,60,61]. Despite a significant decline in travel-related malaria cases in the EU/EEA during 2020 and 2021 due to disruptions in air travel amid the COVID-19 pandemic [2,62], our study identified over 18 cases between 2020 and 2022. This suggests that factors beyond passenger travel volumes may significantly contribute to the occurrence of airport and luggage malaria (e.g. particularly favourable climatic conditions).

The classification of airport malaria is often presented by investigators as a hypothesis following the exclusion of other possible explanations (e.g. recent travel) in addition to a close link to the airport (e.g. living in proximity). As the infected mosquito has to be able to survive at the arrival airport, environmental conditions most likely play a key role and should be considered in any classification, with most airport malaria cases occurring during the warmer months in Europe (June–September). The summer in Europe also coincides with the wet season in some African countries, which may lead to increased mosquito population at the origin airport [63]. Genomic analysis of the clinical sample can provide a better understanding of the phylogenetic properties of the pathogen and geographic origin of the infectious mosquito. For example, Van Bortel et al. predicted the country of origin of two airport malaria cases using genomic data [53]. A better understanding of the country of origin and associated airports with direct flights to Europe could assist in targeting prevention measures.

Nine countries reported cases of airport or luggage malaria in Europe with over 50% of case reports from France. This aligns with data from travel-related malaria where France reported twice as many cases than any country in the EU/EEA, UK and Switzerland [1,64] suggesting close ties with malaria-endemic countries. Further, the high number of travel-associated malaria cases reported in France may be related to a high level of healthcare professional awareness and routine malaria diagnostics and reporting. Most airport malaria cases in France were linked to Paris Charles de Gaulle airport, the airport with the largest air traffic volume in the EU/EEA from malaria-endemic countries. Paris Charles de Gaulle airport was also found to have more direct flights arriving from malaria-endemic countries compared with any other airport in the EU/EEA. Despite the UK’s close historical and economic connections with malaria-endemic countries, only five cases of airport and luggage malaria were reported in the UK (England) from both the literature review and in response to the data call. Like countries in the EU/EEA, the UK has outlined disinsection procedures for aircraft operators in the Health Protection Regulation of 2013 [65]. Further investigation into differences in flight patterns, climatic conditions, disinsection procedures, surveillance and prevention is warranted to better understand differences observed between countries.

To better monitor trends, an improved structured routine surveillance of airport and luggage malaria may be beneficial. Malaria is a notifiable disease at EU level with an established case definition, and data are collected yearly from EU/EEA countries in The European Surveillance System (TESSy) [66]. The collection of importation status allows for classification of cases as travel-related or autochthonous. However, no further information on the route of transmission is collected for locally acquired cases and thus TESSy data do not allow for further classification. While there may be interest in further classifying locally acquired malaria, it's crucial to acknowledge that the annual number of airport and luggage malaria cases is less than 0.2% of imported cases. However, the diagnosis of airport and luggage malaria is often delayed (with a mean of 7.5 days) compared with travel-associated malaria (a median of 3 days for infections with *P. falciparum* [67]). A delayed diagnosis is associated with an elevated risk of severe disease and death, as also shown in the data extracted in this systematic review where the case fatality was 7% compared with 1% in travel-related cases [2]. Indicator-based surveillance would allow for a regional longitudinal perspective on trends over time. To provide an early exchange between EU/EEA countries on airport malaria cases, event-based surveillance (e.g. EpiPulse event-based surveillance) could be a valuable tool [68]. Cases in one EU/EEA country may provide early indication for particularly favourable climatic conditions, which could lead to additional cases at other airports.

A recent modelling study examining the risk of air traffic-induced malaria transmission in central Europe found that, while airport malaria cases can occur through the importation of *P. falciparum* in mosquitoes by air travel, transmission is unlikely to be sustained through indigenous vectors in central Europe [69]. Interestingly, in the mathematical model, the use of aircraft disinsection reduced transmission events towards zero [69]. Aircraft disinsection could particularly decrease the risk for airport personnel working near the cargo hold. The World Health Organization (WHO) outlines aircraft disinsection methods and procedures to prevent importation by aircraft of potential disease vectors. Despite the guidance, there are no standardised procedures in place in the EU/EEA for disinsection in aircrafts [70]. Some countries have regulations in place such as Italy, France, Switzerland and the UK [65,70,71]. The details of the regulations in place differ between countries. For example, the French ministry of health dictates a specific list of countries from which planes must be disinsected [71,72]. Additionally, the regulation dictates the need for quality controls for implementing disinsection [71]. However, overall, very limited information is available about the methods used for disinsection and the results of checks performed. A study conducted between June and September 1995 in France showed that compliance with the legislation to use disinsection in aircrafts significantly increased following enforcement of controls [73]. Further detailed investigation into disinsection practices in the EU/EEA is warranted to better understand the current prevention and control mechanisms and their efficacy, which could further explain country differences observed in this study.

The causative agent for the majority of cases identified in this study (88%) was *P. falciparum*, consistent with travel-related cases where *P. falciparum* constituted 84% in 2021 [2]. Several factors could contribute to this, including variations in the clinical presentation of different *Plasmodium* species, the proximity of *P. falciparum* endemic countries to Europe, along with established direct flight patterns between endemic countries and major international airports in Europe [74]. It is crucial to acknowledge that other *Plasmodium* species may also play a role as the causative agent in airport and luggage malaria cases. However, it is likely that these infections generally exhibit milder forms of the disease and may potentially remain undiagnosed in the early stage (e.g. making them less likely to be classified as airport malaria). Four mixed infections, involving two species of *Plasmodium* in the same patient, were identified in this study. While mixed species infections are not uncommon in endemic countries where both species coexist [75], the simultaneous infection of a single mosquito with two distinct species is less probable. It is important to note that the classification of a case as airport or luggage malaria was not re-evaluated in this study as not enough information was provided to make an accurate and consistent retrospective evaluation. Instead, the classification of the authors/reporters of the data was used for all analyses even if an alternative classification may seem plausible (e.g. two mixed infections cases acquired in a hospital in February).

A primary limitation of this study is the lack of consensus on the definition or criteria for airport or luggage malaria. In this study, the classification by the authors of the original studies was not re-evaluated as it was not feasible given the information provided. Two independent reviewers examined the conclusions of the authors, taking into consideration the most probable classification identified by the original authors and any supporting publication (e.g. reviews or additional publications of the cases at national level). Overall, the quality of the published case reports was good - the description of the actual place of infection, the transmission routes and the case scored well. However, there were cases where the classification made by the authors was questionable. For example, we identified six cases where the authors described a prior stay in an endemic area, but still concluded that airport/luggage malaria was the most probable explanation [11,18,23,38,46,52]. Overall, for many airport malaria cases, there was strong evidence. For luggage malaria, the evidence was less convincing, and was often used as an explanation for a case that could not be otherwise explained. Standardised criteria to support the classification of malaria cases into airport or luggage malaria would be beneficial for the future. Such criteria could for instance include work exposure, travel history and distance from an airport.

A secondary limitation is that the review included cases from 1969 to 2022. We showed that the quality of the reports had significantly improved since 2000, indicating the challenge of conducting a systematic review for such an extended period. Three full text articles could not be retrieved despite best efforts [76-78]. Further, due to data availability issues, the flight pattern analysis conducted for this study only covered 2021 and 2022 and may therefore be influenced by changes to flight patterns following the COVID-19 pandemic.

## Conclusion

While airport and luggage malaria cases are rare, reports in Europe have increased, despite reduced flight volumes during the COVID-19 pandemic. The observed increasing trend highlights the necessity of effective prevention measures and prompts the need for a more structured surveillance of cases in Europe, including a standardised case definition for more consistent case classification. The prolonged diagnosis associated with airport and luggage malaria compared with travel-associated malaria emphasises the urgency to investigate the effectiveness of different prevention measures and improve awareness and case management. Additionally, the prevention measures already in place should be assessed for compliance and effectiveness. For example, standardised procedures in the EU/EEA for disinsection in aircraft in line with the WHO aircraft disinsection methods and procedures, may be of value, considering the increase in connectivity and risk of introduction of various vectors of disease.

## Supplementary Data

498000 Supplementary Material

## Supplementary Data

68000 Supplementary Table S3
