# Supplementary material for: Airport and luggage (Odyssean) malaria in Europe: a systematic review
Source: Euro Surveill. 2024 Oct 10;29(41):2400237. doi: 10.2807/1560-7917.ES.2024.29.41.2400237 (PMC11484919; doi:10.2807/1560-7917.ES.2024.29.41.2400237)
Supplement: Supplementary Material [file 24-00237_HALLMAIER-WACKER_Supplementary_material.pdf]

Supplementary material

Airport and luggage (Odyssean) malaria in Europe: a systematic review

Disclaimer

This supplementary material is hosted by *Eurosurveillance* as supporting information alongside the article 'Airport and luggage (Odyssean) malaria in Europe: a systematic review', on behalf of the authors, who remain responsible for the accuracy and appropriateness of the content. The same standards for ethics, copyright, attributions and permissions as for the article apply. Supplements are not edited by *Eurosurveillance*, and the journal is not responsible for the maintenance of any links or email addresses provided therein.

Supplementary methods

List of variables collected in data call

Cases identified through the literature were included in the data sheet to allow the countries to check the published data and supplement any additional data. The following variables were included in the data sheet sent to the countries: Case category, case definition used (Yes/No), case definition provided, exact date of infection provided (Yes/No), date of infection, date of symptom onset, date of admission or date of first seeking treatment, date of diagnosis, time to diagnosis in days, city/region place of infection, associated airport, distance place of infection to airport (km), country place of infection, place of diagnosis (city), place of diagnosis (country), age of case (years), age category (0-10, 11-20, 21-30, 31-40, 41-50, 51-60, 61-70, 71-80, 81+), gender of case, outcome (dead/recovered), relevant occupation (Yes/No), relevant occupational activity, recent travel history to a malaria endemic area (Yes/No), blood transfusion or drug abuse (Yes/No), malaise (Yes/Not specified), fever (Yes/Not specified, headache (Yes/Not specified, muscle stiffness/pain (Yes/Not specified, chills (Yes/Not specified, sweats (Yes/Not specified), nausea and vomiting (Yes/Not specified), body aches (Yes/Not specified, abdominal pain/digestive problems (Yes/Not specified , Other symptoms/signs/syndromes, microscopy (such as blood smear) (Positive, Negative, Not tested), antigen tests (Rapid Diagnostic Test) (Positive, Negative, Not tested), molecular diagnosis (nucleic acid amplification test such as PCR) (Positive, Negative, Not tested), antibody tests (Indirect Fluorescent Antibody test (serology)) (Positive, Negative, Not tested), Drug resistance test (Positive, Negative, Not tested), other tests, species of plasmodium, haematological parameters presented (Yes/No), describe the links between cases, possibility of local vector discussed (Yes/No), possible country imported mosquito, and link to airport (1 = Occupation directly linked to airport, 2 = Residence or working near the airport, 3 = Visited airport (to take a flight, stay in a hotel at the airport, or picking someone up), 4 = Not direct exposure to airport - the infected mosquito is brough back in the luggage of a relative arriving from an endemic country, 5 = Not direct exposure to airport - other)

Flight pattern analysis

Flight traffic data from January 2021 to December 2022 collected by EUROCONTROL was used to analyse destinations of flights departing from malaria endemic countries. Countries were classified as malaria endemic countries for this study if the incidence of > 70 *P. falciparum* cases per 1 000 population in 2020 (as reported by the Malaria Atlas Project Data Platform (https://malariaatlas.org/)). We choose to focus only on *P. falciparum* as the vast majority of cases identified in the systematic review were caused by this species. Seasonal differences in incidence were not considered in this analysis, as *Plasmodium* circulation is expected to occur at some level all year round in endemic countries. Destination airports in Switzerland and United Kingdom as well as airports in EU oversea territories and islands were excluded from the analysis. All analyses were performed using the tidyverse and ggalluvial package in R (V.4.0.2).

Supplementary figures

Figure S1: Distribution of age and sex of airport and luggage malaria for A) all reported case reports B) case reports in individuals working at the airport.

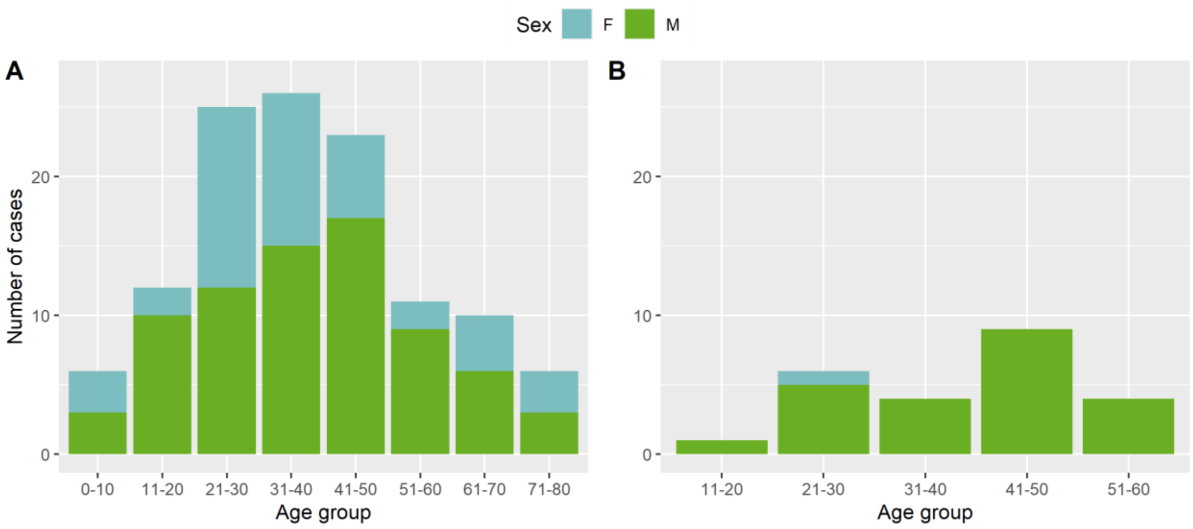

Figure S2. Map of luggage malaria cases by airports* and country

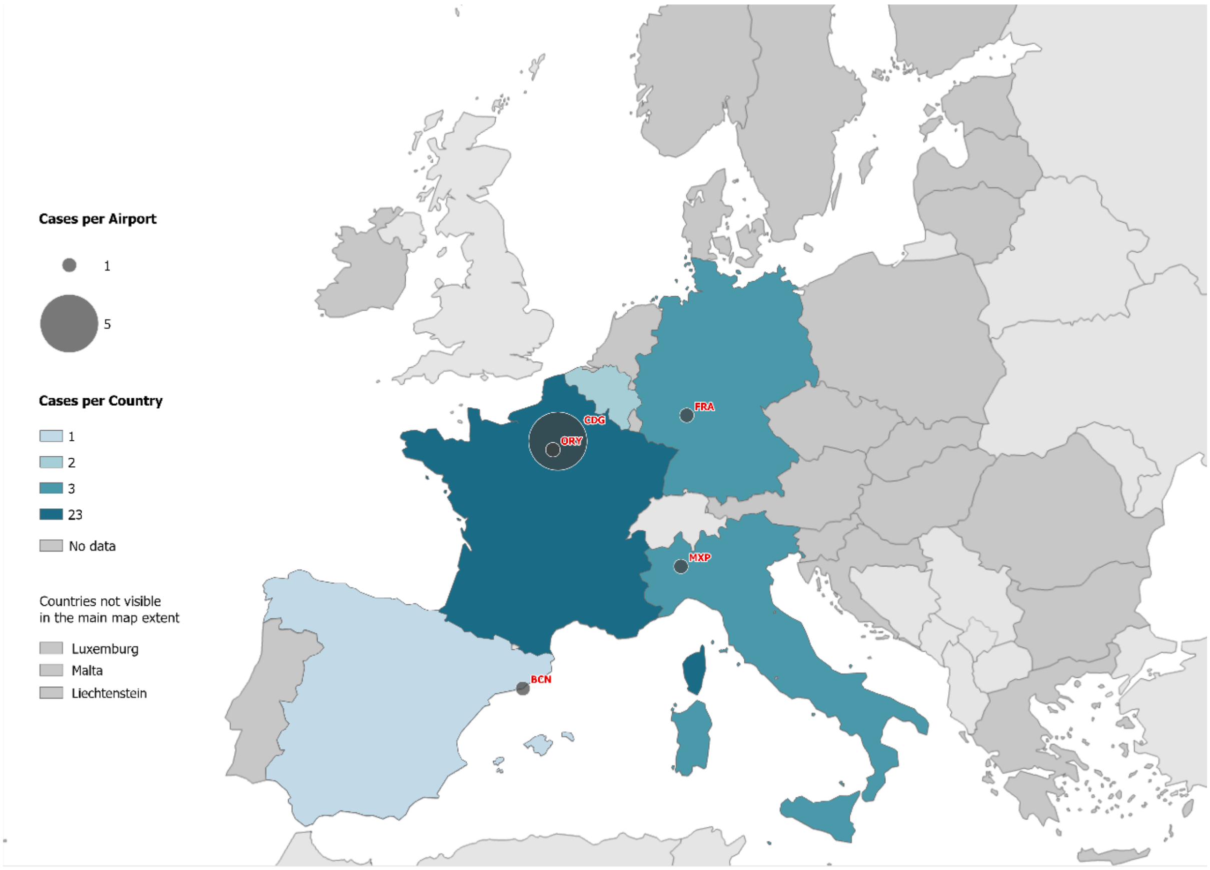

* Barcelona Airport (BCN, n = 1), Charles de Gaulle Airport (CDG, n = 5), Frankfurt Airport (FRA, n = 1), Milan Malpensa Airport (MXP, n = 1), Paris Orly Airport (ORY, n = 1)

Figure S3: Direct flights from endemic countries (>70 *P. falciparum* cases per 1 000 population in 2020) to top 5 destination airports in the EU/EEA per month in 2021 and 2022. The following copyright applies to the flight data used in this figure: © EUROCONTROL 2024.

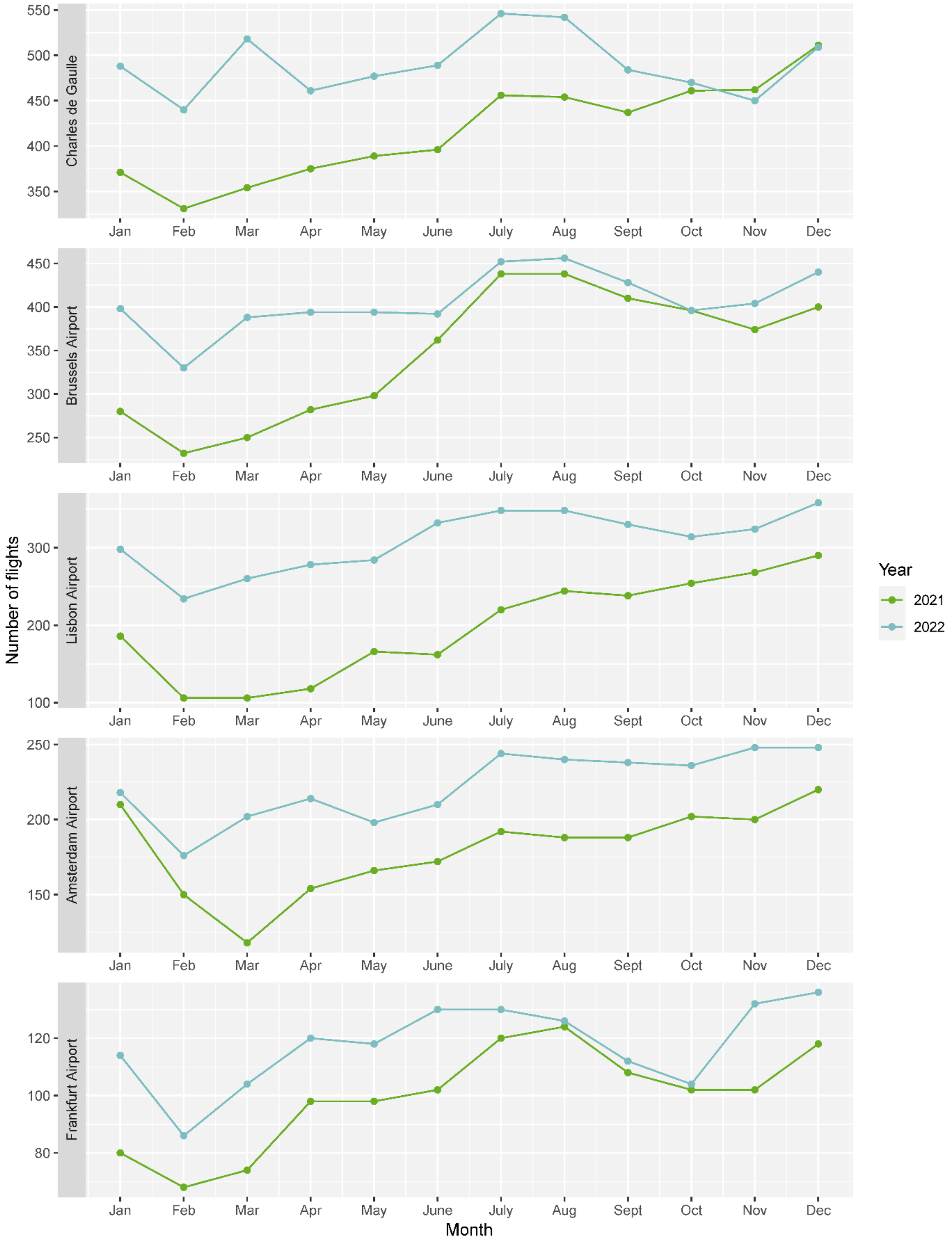

Table S1: Search strings based on defined search strategy

Pubmed

Search Number	Search String	Hits	Date
#1	malaria[mesh] OR malaria*[tiab] OR plasmodium[mesh] OR plasmodi*[tiab] OR falciparum[tiab] OR (plasmodi* w3 ovale[tiab]) OR (p w3 ovale[tiab]) OR vivax[tiab] OR knowlesi[tiab]	123 981	01/01/2024
#2	airport[mesh] OR airport*[tiab] OR luggage*[tiab] OR suitcase*[tiab] OR baggage*[tiab] OR airplane*[tiab] OR aircraft*[tiab] OR airline*[tiab] OR plane*[tiab]	194,709	01/01/2024
#3	#1 AND #2	316	01/01/2024

Embase.com

Search Number	Search String	Hits	Date
#1	'malaria'/de OR 'malaria*':ab,ti OR 'plasmodium'/de OR 'plasmodi*':ab,ti OR 'falciparum':ab,ti OR 'plasmodi* NEXT/3 ovale':ab,ti OR 'p NEXT/3 ovale':ab,ti OR 'vivax':ab,ti OR 'knowlesi':ab,ti	158 340	01/01/2024
#2	'airport'/de OR 'airport*':ab,ti OR 'luggage*':ab,ti OR 'suitcase*':ab,ti OR 'baggage*':ab,ti OR 'airplane*':ab,ti OR 'aircraft*':ab,ti OR 'airline*':ab,ti OR 'plane*':ab,ti	204 638	01/01/2024
#3	#1 AND #2	408	01/01/2024

Scopus

Search Number	Search String	Hits	Date
#1	TITLE-ABS(malaria* OR plasmodi* OR falciparum OR (plasmodi* W/3 ovale) OR (p W/3 ovale) OR vivax OR knowlesi)	140 031	01/01/2024
#2	TITLE-ABS(airport* OR luggage* OR suitcase* OR baggage* OR airplane* OR aircraft* OR airline* OR plane*)	1 496 680	01/01/2024
#3	#1 AND #2	426	01/01/2024

Ovid Medline

Search Number	Search String	Hits	Date
#1	malaria.sh OR malaria*.tw OR plasmodium.sh OR plasmodi*.tw OR falciparum.tw OR (plasmodi* adj3 ovale).tw OR (p adj3 ovale).tw OR vivax.tw OR knowlesi.tw	123 011	01/01/2024

#2	airport.sh OR airport*.tw OR luggage*.tw OR suitcase*.tw OR baggage*.tw OR airplane*.tw OR aircraft*.tw OR airline*.tw OR plane*.tw	194 500	01/01/2024
#3	#1 AND #2	314	01/01/2024

EBSCO open dissertations

Search Number	Search String	Hits	Date
#1	malaria* OR plasmodi* OR falciparum OR (plasmodi* N/3 ovale) OR (p N/3 ovale) OR vivax OR knowlesi	2 557	01/01/2024
#2	airport* OR luggage* OR suitcase* OR baggage* OR airplane* OR aircraft* OR airline* OR plane*	24 341	01/01/2024
#3	#1 AND #2	10	01/01/2024

OpenGrey

Search Number	Search String	Hits	Date
#1	(malaria* OR plasmodi* OR falciparum OR (plasmodi* NEAR/3 ovale) OR (p NEAR/3 ovale) OR vivax OR knowlesi) AND (airport* OR luggage* OR suitcase* OR baggage* OR airplane* OR aircraft* OR airline* OR plane*)	4	01/01/2024

Table S2: Quality assessment tool adapted from the National Institutes of Health Quality Assessment Tool used to assess methodological quality*.

Criteria	Scoring of the criteria (score of 0 or 1 given)
**1.** Was the study question or objective clearly stated?	0=No clear study question or objective was stated<br>1= A study question or objective was clearly stated
**2.** Was the case definition clearly described?	0= No definition of airport or luggage malaria was reported<br>1 = a definition of airport or luggage malaria was reported or referred to
**3**. Where the persons clearly described?	0= No clear description of the persons was reported, 1 = general characteristics of the persons were clearly reported (e.g., age, gender)
**4.** Was the place of infection described or were epidemiological circumstances identified?	0= No place of infection or epidemiological circumstances were reported<br>1= The (suspected) place of infection and other relevant epidemiological information were clearly reported (e.g., place of occupation, travel history, etc.)
**5.** Was the clinical history of the persons clearly described?	0= No clinical history was reported<br>1= Relevant clinical history was reported
**6.** Were diagnostic methods clearly described?	0= No diagnostic methods were described<br>1= Diagnostic method(s) used for the detection of malaria were described and the results were clearly reported
**7.** Were the possible routes of transmission clearly discussed and evaluated?	0= No routes of transmission were discussed or evaluated<br>1= Possible transmission routes were assessed and evaluated
Additional question for multiple cases described in a single manuscript:	
**8.** Were multiple cases within the same cluster presented consistently along the same criteria and case definition, and was an epidemiologically link between them accessed?	0= Multiple cases were not presented according to the same criteria and the epidemiological link was not accessed<br>1= Multiple cases were presented consistently and a possible epidemiological link between the cases was investigated

*The following numeric ranking was used: studies with a single case report: 1–3 = Poor, 4–5 = Fair, 6 –7 = Good; studies with multiple case reports: 1–4 = Poor, 5–6 Fair, 7–8 Good

Table S4: Top departure country in the malaria endemic area countries (>70 *P. falciparum* cases per 1 000 population in 2020) by number of direct flights with destination in an EU/EA country in 2021 and 2022. The following copyright applies to the flight data used in this table: © EUROCONTROL 2024.

Country	2021	2022
Senegal	3580	4371
Nigeria	2719	3145
Ghana	1914	2005
Angola	1212	2452
Ivory Coast	1504	1513
Uganda	1081	1340
Niger	1228	1340

Table S5: Top destination airports in the EU/EEA by number of direct flights arriving from malaria endemic countries (>70 *P. falciparum* cases per 1 000 population in 2020) in 2021 and 2022. Data in paratheses describe the number of direct flights to the country in which the airport is located. The following copyright applies to the flight data used in this table: © EUROCONTROL 2024.

Airport (Country)	2021	2022
Paris Charles de Gaulle Airport (FR)	4997 (7280)	5874 (8102)
Brussels Airport (BE)	4160 (4686)	4872 (5296)
Humberto Delgado Airport –Lisbon airport (PT)	2358 (2576)	2358 (3916)
Amsterdam Airport -Schiphol (NL)	2160 (2212)	2672 (2688)
Frankfurt am Main Airport (DE)	1194 (2105)	1412 (2251)
Adolfo Suárez Madrid Airport (ES)	756 (1838)	1066 (2490)
Paris–Le Bourget Airport (FR)	730 (7280)	548 (8102)
Josep Tarradellas Barcelona Airport (ES)	438 (1838)	806 (2490)
